# On the Effect of Hydrogen on the Low-Temperature Elastic and Anelastic Properties of Ni-Ti-Based Alloys

**DOI:** 10.3390/ma10101174

**Published:** 2017-10-13

**Authors:** Konstantin Sapozhnikov, Joan Torrens-Serra, Eduard Cesari, Jan Van Humbeeck, Sergey Kustov

**Affiliations:** 1 Ioffe Institute, Politekhnicheskaya 26, 194021 St. Petersburg, Russia; 2 ITMO University, Kronverkskiy 49, 197101 St. Petersburg, Russia; sergey.kustov@uib.es; 3 Departament de Física, Universitat de les Illes Balears, Cra Valldemossa km 7.5, E 07122 Palma de Mallorca, Spain; j.torrens@uib.es (J.T.-S.); eduard.cesari@uib.cat.es (E.C.); 4 Department MTM, Katholieke Universiteit Leuven, Kasteelpark Arenberg 44, B-3001 Leuven, Belgium; jan.vanhumbeeck@kuleuven.be

**Keywords:** shape memory alloy, acoustic properties, internal friction, lattice defects, hydrogen

## Abstract

Linear and non-linear internal friction and the effective Young’s modulus of a Ni_50.8_Ti_49.2_ alloy have been studied after different heat treatments, affecting hydrogen content, over wide ranges of temperatures (13–300 K) and strain amplitudes (10^−7^–10^−4^) at frequencies near 90 kHz. It has been shown that the contamination of the alloy by hydrogen strongly affects the internal friction and Young’s modulus of the martensitic phase. Presence of hydrogen gives rise to a non-relaxation internal friction maximum due to a competition of two different temperature-dependent processes. The temperature position and height of the maximum depend strongly on the hydrogen content. We conclude that many of the internal friction peaks, reported earlier for differently treated Ni-Ti-based alloys, had the same origin as the present maximum.

## 1. Introduction

Ni-Ti-based alloys represent the most important family of shape memory alloys for applications. Anelastic properties of Ni-Ti-based alloys have attracted considerable attention, both for the microstructural characterization of the alloys and for their application as high-damping materials [1]. However, the bulk of studies have been performed in the temperature range around phase transformation temperatures. Anelastic properties of martensitic phases have attracted less attention, especially at low temperatures. Nevertheless, a variety of maxima were reported in the temperature spectra of the internal friction (IF). The so-called ‘200 K’ relaxation IF peak was first observed by Hasiguti and Iwasaki [2] and then by many other researchers [1]. It has been attributed to an interaction of twin boundaries with hydrogen [3,4]. Another IF peak around 100 K was reported and ascribed to a dislocation relaxation by Tirbonod and Koshimizu [5]. Numerous peaks were found by Mazzolai et al. at temperatures between 40 and 240 K: P_TWM_ [6,7,8,9,10], P_d_ [6,7], P_150K_ [11,12,13], P_200K’_ [13], P_50K_ [13], and also several peaks for hydrogen-doped samples [13]. Their origin is still unclear and calls for further studying.

Recently, the effect of intentional hydrogenation of Ni-Ti-based alloys on their anelastic properties was actively studied. IF data for H-loaded samples were compared with those for “H-free” samples [3,4,14,15,16]. On the other hand, Fan et al. [3,4] have shown that hydrogen is unintentionally introduced during conventional heat treatment (water quenching after high-temperature annealing in argon-filled [3] or vacuum-sealed [4] quartz tubes) by the chemical reaction of residual water in quartz tubes with Ti at high temperatures. This raises doubts as to whether the “H-free” samples are really free from H and H-related anelastic effects.

The present paper is devoted to investigations of low-temperature anelastic properties of a Ni-Ti alloy after different heat treatments. First, measurements were carried out in an as-received state with low hydrogen content and after conventional heat treatments contaminating the alloy by hydrogen. Then, a series of long-term high-temperature anneals in vacuum (under continuous pumping) was performed with measurements in between the annealing steps to study the effect of dehydrogenation. Such a procedure allows us to shed more light on the effect of hydrogen contamination during conventional heat treatments of Ni-Ti-based alloys on their elastic and anelastic properties.

## 2. Materials and Methods

The Ni_50.8_Ti_49.2_ alloy was supplied in a cold-worked state (cold-drawn wire 2.8 mm in diameter, strain of about 40%). Rod-shaped samples for acoustic measurements were spark cut and mechanically polished. One sample was tested in the as-received (AR) state. Another one was subjected to a sequence of heat treatments: (1) vacuum quenching (VQ); (2) water quenching (WQ); and, (3) four consecutive vacuum annealings (VA1–VA4). The VQ and WQ treatments included annealing for 2 h at 1273 K in a vacuum-sealed quartz tube followed by quenching into water with (WQ) or without (VQ) breaking the tube. The VA1 treatment consisted in annealing for 3 h at 870 K and for 1 h at 930 K under a residual gas pressure P ~ 1 Pa. The VA2/VA3/VA4 treatments involved annealing for respectively 8/16/34 h at 950 K under P ~ 10^−3^ Pa. The annealing temperatures were chosen to be higher than the temperature ranges of hydrogen desorption from Ni-Ti alloys reported in the literature [17,18,19].

The IF and effective Young’s modulus of the samples were measured in the temperature range 13–300 K by means of the resonant piezoelectric composite oscillator technique [20] using longitudinal oscillations at frequencies near 90 kHz. The logarithmic decrement *δ*, defined as *δ* = *∆W/2W*, where *∆W* is the energy dissipated per cycle and *W* is the maximum stored vibrational energy, was used as a measure of the IF. A computer-controlled setup [21] enabled us to measure continuous temperature spectra of the IF and Young’s modulus at two values of oscillatory strain amplitude. A low value of strain amplitude (10^−6^) was selected to fall within the linear (strain amplitude-independent) range of the IF and Young’s modulus, whereas the second one was sufficiently high to observe non-linear (strain amplitude-dependent) effects (in the present paper we will report only amplitude-independent temperature spectra). The samples were cooled/heated in a He atmosphere in an Oxford close-loop cryostat at a heating/cooling rate of about 2 K/min. In some thermal cycles, the strain amplitude dependence of the IF and modulus defect was measured at different temperatures for the strain amplitudes between 2 × 10^−7^ and 10^−4^. The temperature was stabilized before each measurement. During measurements of the strain amplitude dependence, the oscillatory strain amplitude was first increased with a preset step from the lowest value and then decreased in a reverse sequence (forward and reverse runs).

The temperatures of the B2 ↔ B19’ martensitic transformations were determined from differential scanning calorimetry and electrical resistance data. The calorimetry data were obtained using Mettler-Toledo DSC823 calorimeter. The four-wire alternating current impedance measurements were performed at frequency of 686 Hz and cooling/heating rate of 2 K/min. The real part of the impedance R was measured by means of a lock-in amplifier.

Hydrogen content in variously treated samples was quantified by means of the vacuum hot extraction method. An industrial hydrogen analyzer AV-1 was used with mass-spectrometric registration of the time dependence of hydrogen flux from samples heated in vacuum [22]. The analyzer was calibrated on certified hydrogen containing samples of an aluminium alloy with the error of the certified value of hydrogen concentration of 6%. Hydrogen was extracted from samples in two successive steps at temperatures of 803 and 1073 K, under a working pressure of 10^−4^ Pa.

## 3. Results

### 3.1. Characterization of Martensitic Transformations Using Calorimetry and Resistance Data

**Figure 1:**
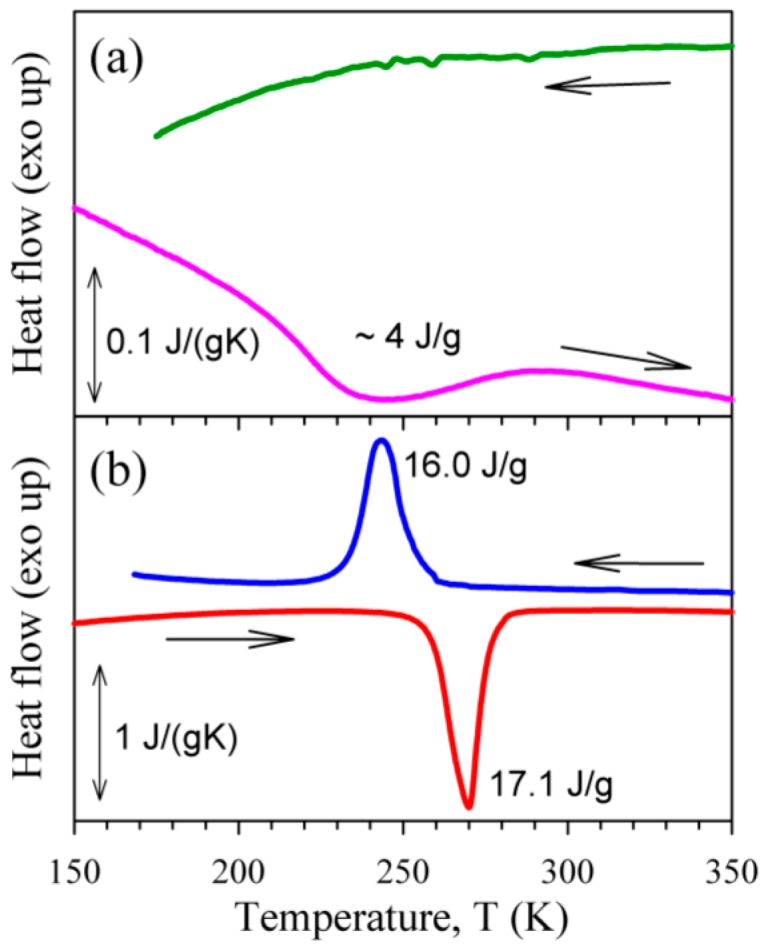
Calorimetry scans for the as-received (**a**) and water-quenched (**b**) Ni_50.8_Ti_49.2_ samples.

**Figure 2:**
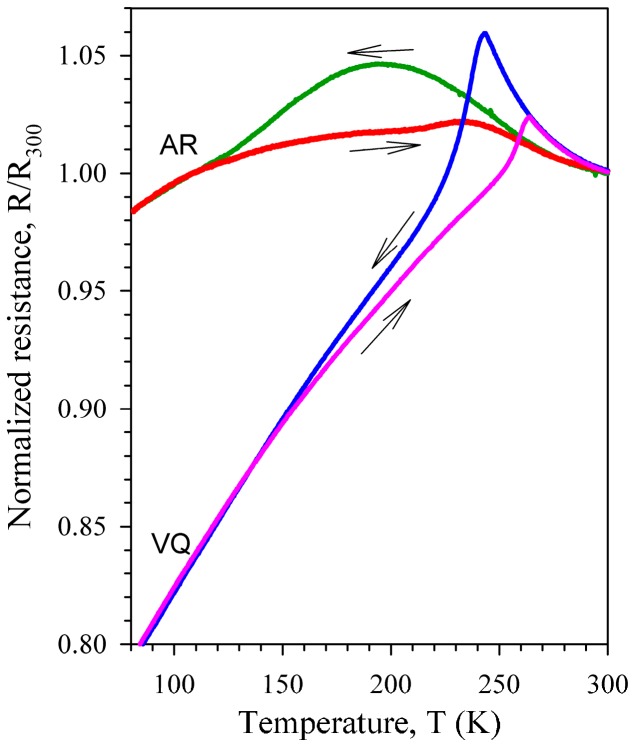
Temperature dependence of resistance for the as-received (AR) and vacuum-quenched (VQ) Ni_50.8_Ti_49.2_ samples. Data are normalized to the resistance values at 300 K.

Figure 1 shows calorimetry scans for the AR (a) and WQ (b) samples. It is seen that the martensitic transformation in the AR state is only partial and very diffuse. The forward martensitic transformation is hardly discernible in the calorimetry data for the AR state, whereas the reverse martensitic transformation is more pronounced allowing an estimation of the total heat at about 4 J/g (around 20% of that for the WQ state). The normalized resistance data for the AR and VQ samples are depicted in Figure 2. Here, again the partial and diffuse character of the martensitic transformation in the AR state is evident. The start and finish temperatures of the martensitic and reverse transformations were evaluated from the calorimetry and resistance data as M_s_ = 192/253/241 K, M_f_ = 120/233/225 K, A_s_ = 210/258/252 K, A_f_ = 280/281/265 K for the AR/WQ/VQ samples, respectively.

### 3.2. Hydrogen Content Evaluation

Results of the hydrogen content evaluation in differently treated samples (AR, WQ, and VA4) by means of the vacuum hot extraction method are summarized in Table 1. It is seen that hydrogen is present in all of the samples and its content varies considerably with heat treatment. The total extracted hydrogen content in the WQ state is about four times higher as compared to the original AR state. The dehydrogenation treatment (four consecutive vacuum annealings) of the WQ sample reduces the total extracted hydrogen content by about an order of magnitude. It is important to note that the effect of the heat treatments is much stronger for the hydrogen fraction extracted at 803 K, because hydrogen is preferably accumulated in deep traps in the samples with low hydrogen content (AR and VA4), whereas weakly bound states of hydrogen prevail in the WQ sample with increased hydrogen content.

### 3.3. Temperature Dependence of the Internal Friction and Young’s Modulus

**Figure 3:**
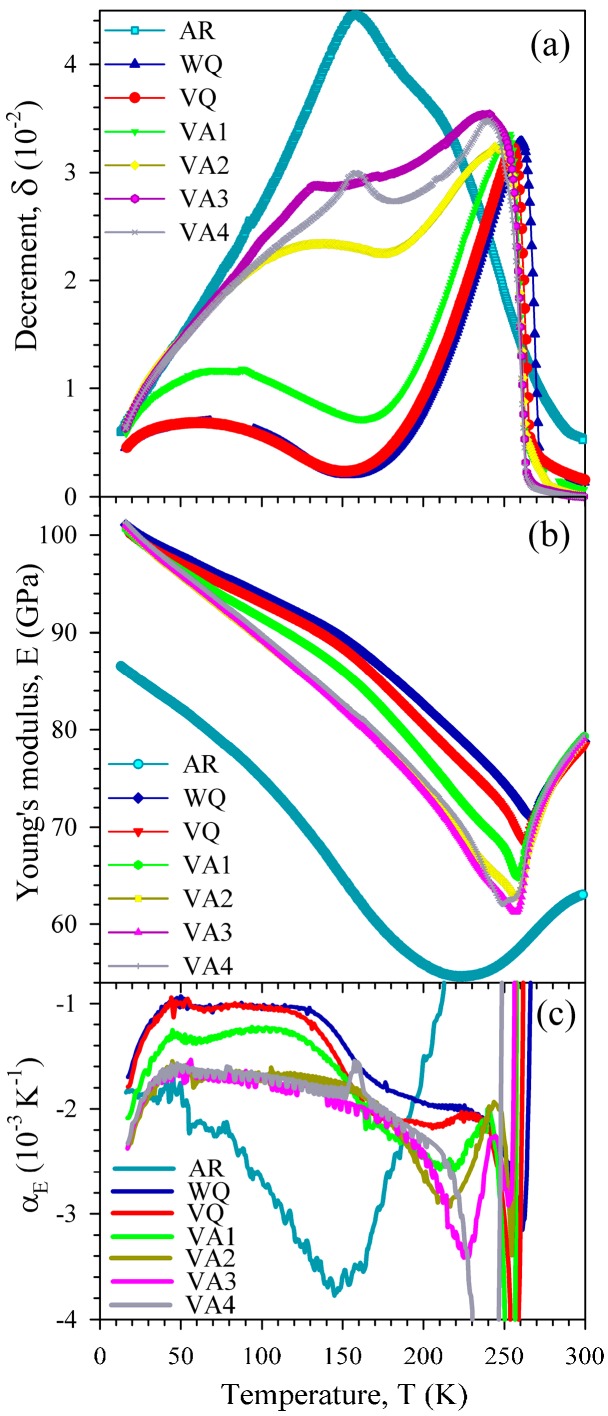
Temperature dependence of the decrement (**a**), effective Young’s modulus (**b**) and temperature coefficient of the Young’s modulus (**c**) of Ni_50.8_Ti_49.2_ samples measured on heating at strain amplitude of 10^−6^ in the as-received state (AR) and after different heat treatments (water quenching (WQ), vacuum quenching (VQ), and vacuum annealings (VA1-VA4), see text for details).

Figure 3 shows the temperature spectra of the amplitude-independent IF (a) and effective Young’s modulus (b) measured on heating for samples in the AR state and after different heat treatments. A high IF maximum at about 160 K and a shoulder on its high-temperature side (around 210 K) are seen for the AR state. Two spectra after conventional heat treatments (VQ and WQ) are very similar and exhibit two IF maxima: (1) a broad maximum near 60 K; and, (2) a high maximum at temperatures of the reverse martensitic transformation, accompanied by a minimum of the effective Young’s modulus. The only difference between these spectra is in the shift of the martensitic transformation maximum by a few degrees, probably, because of the different quenching rate. The low-temperature IF maximum does not depend on the quenching conditions (VQ or WQ). Vacuum annealing strongly affects the IF of the alloy. The IF of the martensite becomes successively higher (except for the VA4 treatment), and the low-temperature IF maximum is progressively shifted from around 60 to 160 K.

The values of the Young’s modulus for the AR sample are notably lower than those after all of the heat treatments (see Figure 3b). A smooth Young’s modulus minimum for the AR sample is another important peculiarity, which reflects the diffuse character of the martensitic transformation in the AR state. The Young’s modulus curves for the heat treated states nearly coincide at the lowest temperatures and in the austenitic state. The effective Young’s modulus of the martensitic phase is the highest after the conventional heat treatment (WQ). The VQ curve differs only slightly from the WQ curve. Vacuum annealing lowers the effective Young’s modulus of the martensitic phase over nearly the entire temperature range. The most pronounced effect is observed just after the first annealing (VA1). A notable change of the temperature coefficient of the Young’s modulus, *α_E_* = (*1/E*_0_) × (*dE/dT*), with *E_0_* taken as the Young’s modulus value at T = 300 K, at low temperatures (below 150 K) as a result of vacuum annealing is the most intriguing effect. Figure 3c shows the temperature dependence of *α_E_*, calculated for all of the curves shown in Figure 3b. One can see that the absolute values of the temperature coefficient increase considerably at low temperatures after the VA1 and VA2 anneals. Another important point is that the absolute values of the temperature coefficient pass through a flat minimum between 50 and 150 K (except for the AR state).

**Figure 4:**
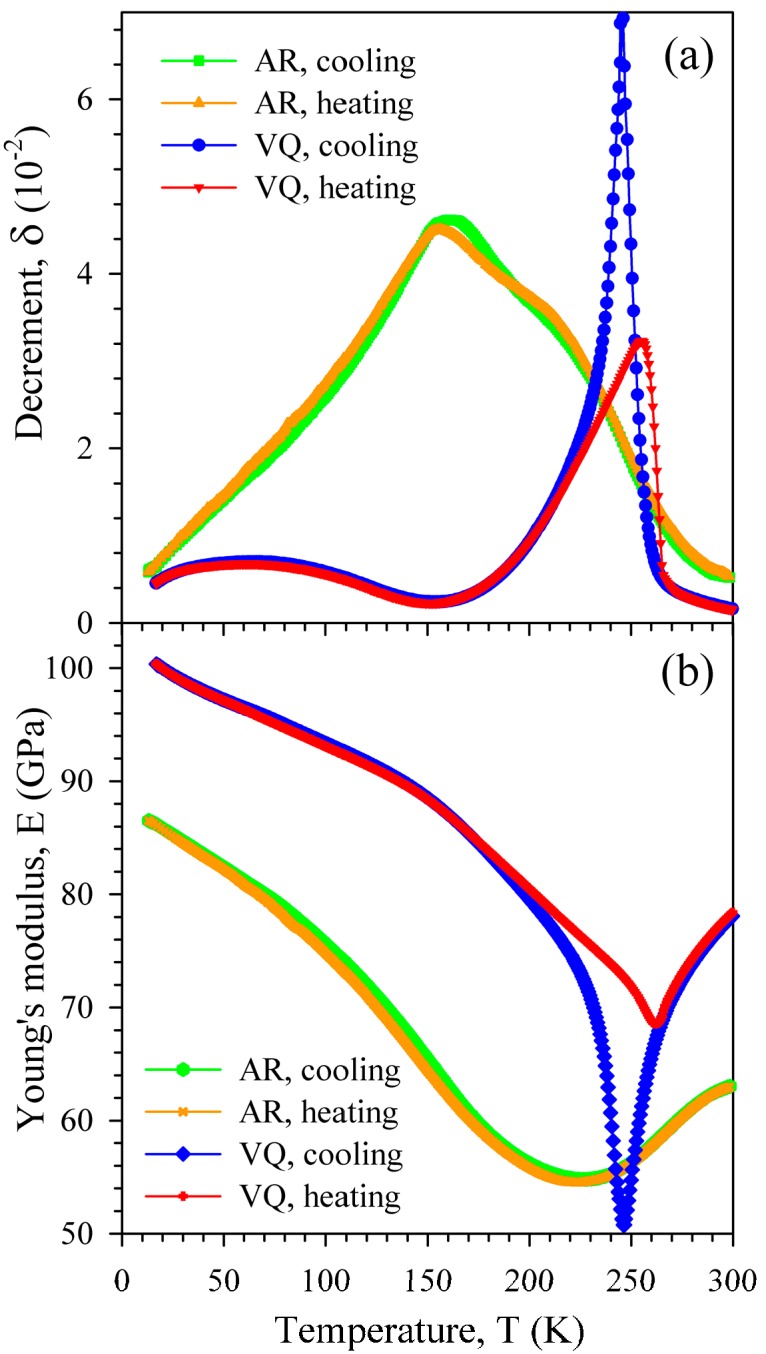
Temperature dependence of the decrement (**a**) and effective Young’s modulus (**b**) of the as-received (AR) and vacuum-quenched (VQ) Ni_50.8_Ti_49.2_ samples measured in a cooling-heating cycle at strain amplitude of 10^−6^.

Temperature dependences of the amplitude-independent IF (a) and effective Young’s modulus (b) of the AR and VQ samples measured in a cooling-heating cycle are represented in Figure 4 to show the details of temperature hysteresis (difference between cooling and heating runs). The dependences for the VQ sample display temperature hysteresis in the martensitic transformation temperature range. Similar temperature hysteresis was observed after other heat treatments. In contrast to the heat-treated sample, the dependences for the cold-worked AR sample demonstrate only minor temperature hysteresis.

### 3.4. Strain Amplitude Dependence of the Internal Friction

**Figure 5:**
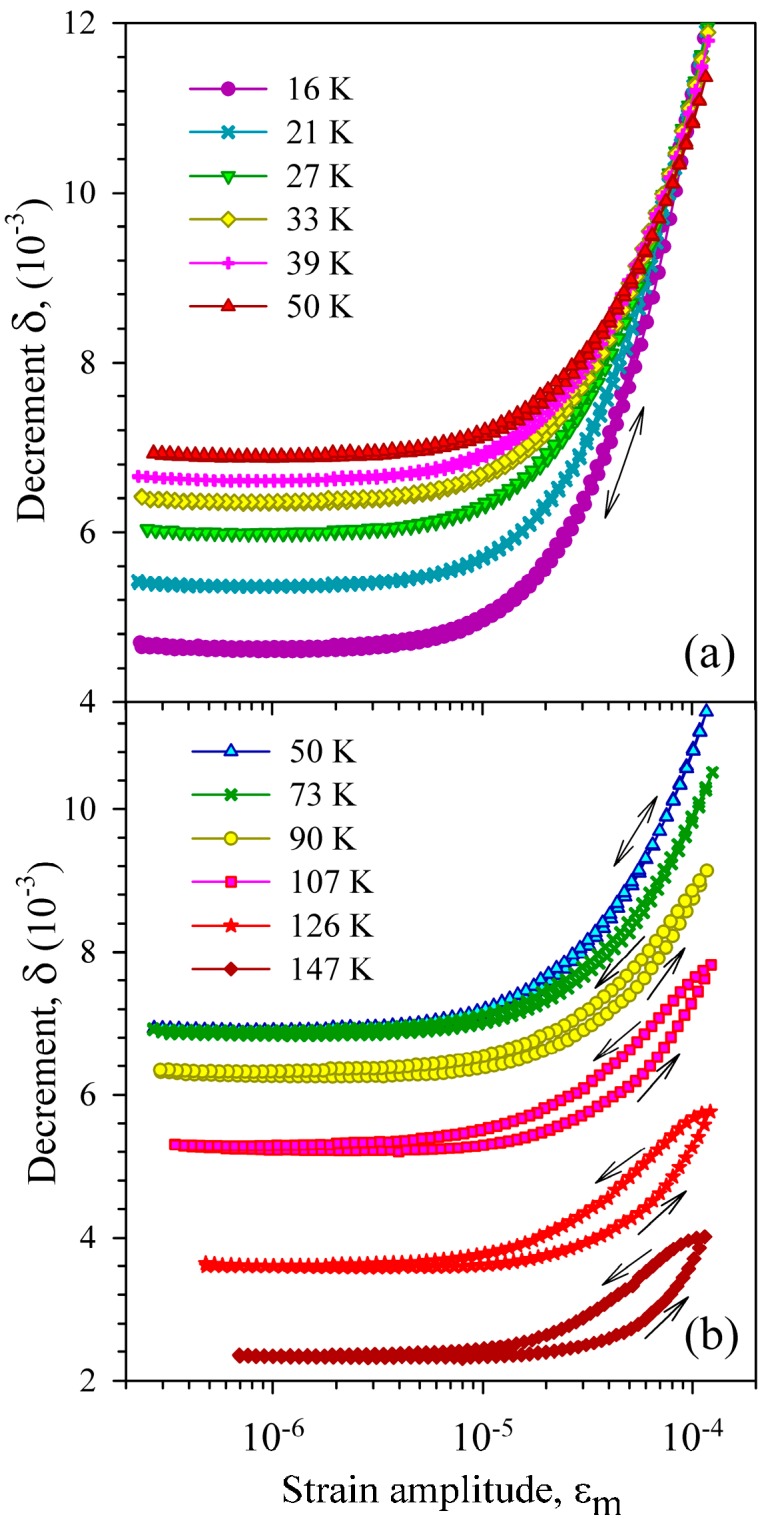
Strain amplitude dependence of the decrement of a Ni_50.8_Ti_49.2_ sample, subjected to vacuum quenching (VQ), measured at different temperatures during heating from 16 K.

**Figure 6:**
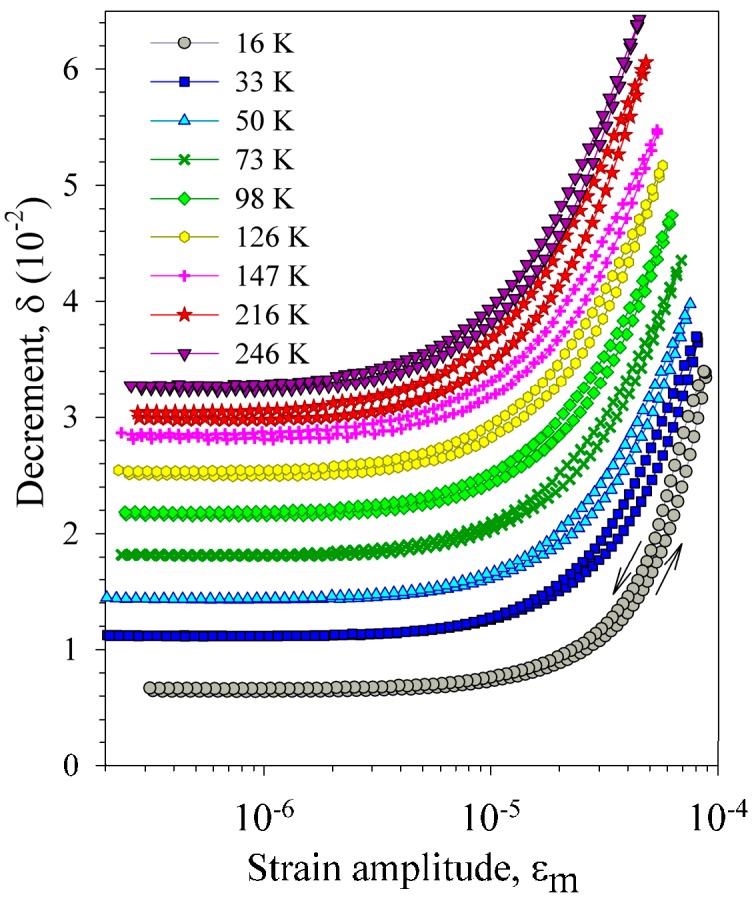
Strain amplitude dependence of the decrement of a Ni_50.8_Ti_49.2_ sample, subjected to vacuum annealing No. 4 (VA4), measured at different temperatures during heating from 16 K.

Figure 5 and Figure 6 display the strain amplitude dependence of the IF measured for the heat-treated sample before (VQ treatment, Figure 5) and after (VA4 treatment, Figure 6) dehydrogenation. It is seen that dehydrogenation affects strongly both the magnitude and the temperature dependence of the amplitude-dependent IF. The amplitude-dependent IF is much higher after dehydrogenation and its temperature dependence reverses sign from negative to positive (a decrease of the amplitude-dependent IF with temperature rise is replaced by an increase). Dehydrogenation also affects the strain amplitude hysteresis of the IF. The latter effect is revealed as the difference between forward and reverse runs of the dependences: the IF is higher for decreasing strain amplitudes than for increasing ones. Before dehydrogenation the amplitude hysteresis is hardly distinguishable for measurements at the low-temperature slope of the IF maximum (Figure 5a) and becomes pronounced at the high-temperature slope (Figure 5b). After dehydrogenation only minor amplitude hysteresis persists, scarcely affected by temperature (Figure 6).

**Figure 7:**
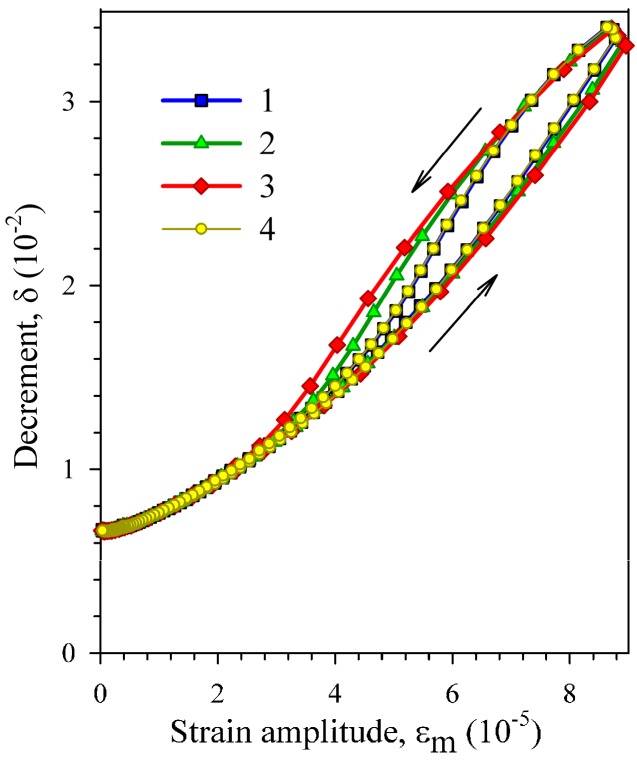
Strain amplitude dependence of the decrement of a Ni_50.8_Ti_49.2_ sample, subjected to vacuum annealing No.4 (VA4), measured at 16 K in four consecutive runs with different measurement steps: 10% (1,4), 20% (2), 30% (3).

The observed amplitude hysteresis of the IF is reversible (reproducible) in consecutive measurements and tends to be more pronounced with increasing measurement step, as is illustrated by Figure 7. One can see that the first and fourth curves, measured with the same measurement step of 10% (of the current value of strain amplitude), coincide perfectly. The second and third curves, measured with the step of 20% and 30%, respectively, exhibit expanding amplitude hysteresis.

## 4. Discussion

The anelasticity of the martensitic phases of Ni-Ti-based alloys is usually attributed to the motion of twin boundaries (see, for example, References [1,13]), though dislocation mechanisms (involving twin [5,23] or bulk [6] dislocations) are also occasionally invoked. The data presented here do not permit further refinement; therefore, twin boundaries will be considered hereafter as sources of the IF of the martensitic phase, unless otherwise stated.

The partial and diffuse character of the martensitic transformation in the cold-worked AR state is not surprising. Cold-working of Ni-Ti-based alloys is known to form the microstructures, such as nanocrystalline and amorphous ones, which substantially suppress the martensitic transformation. According to Reference [24], 40% cold rolling of a Ni_50.2_Ti_49.8_ ribbon produced amorphization as well as stabilization of the B2 structure in textured nanograins with the average size of about 20 nm. The temperature dependences of the IF and Young’s modulus for the cold-worked AR sample (see Figure 4) reproduce in detail those observed earlier for the cold-worked Ni_50.8_Ti_49.2_ alloy in a low-kHz frequency range: a single IF maximum at about 150 K and only slight temperature hysteresis (see Figure 6 in Reference [6] and Figure 3 in Reference [11]). That is why we believe that this is the same frequency-independent IF maximum. The lack of a frequency shift of the IF maximum indicates that it is not relaxational, despite of the maximum absolute *α_E_* values in the vicinity of this maximum. The slight temperature hysteresis of the IF and Young’s modulus contrasts with the extended temperature hysteresis of the martensitic transformation testified by the resistivity data (Figure 2). This indicates that neither the martensitic transformation nor the specific structural defects of the martensitic phase (twins) appreciably contribute to rather high amplitude-independent IF of the cold-worked AR sample. Hence, grain boundaries and/or bulk dislocations are to be considered as the defects responsible for the amplitude-independent IF of the cold-worked sample. We note here that a rather high level of the amplitude-independent IF was observed earlier in a number of ultra-fine grained metals and was attributed to the motion of grain boundary dislocations [25], intragranular dislocations [26], or to grain boundary sliding [27].

The drastic changes in the low-temperature IF and effective Young’s modulus as a result of heat treatments can be associated primarily with changes in the hydrogen content. After the conventional heat treatment (WQ and VQ) contaminating the alloy by hydrogen, the low-temperature IF is rather low and the IF maximum is observed at about 60 K. Subsequent dehydrogenation treatments cause the IF of the martensite to increase over the entire temperature range and shift the IF maximum to about 160 K. The coincidence of the WQ and VQ curves at low temperatures shows that breaking the quartz tube or not during quenching has no effect on the hydrogen content in agreement with the results of Reference [3]. Thus, the low-temperature IF maximum can be attributed to an interaction of twin boundaries with hydrogen. However, it should be distinguished from the ‘200 K’ relaxation IF peak, also attributed to an interaction of twin boundaries with hydrogen [3,4]. Firstly, the ‘200 K’ relaxation IF peak cannot be observed at our frequency, because its estimated temperature position corresponds to the austenitic phase state. Secondly, its temperature position depends only slightly on hydrogen content and demonstrates the opposite trend: the peak shifts to higher temperatures with increasing hydrogen content [16].

The Young’s modulus data for the heat-treated sample show that the low-temperature IF maximum is not of a relaxational nature: the minimum of the absolute values of temperature coefficient in the vicinity of the IF maximum is contrary to what is characteristic of relaxation. Such behaviour of the effective elastic modulus on heating is usually ascribed to a pinning (annealing, recovery) stage (see, for example, References [28,29,30]). The pinning of twin boundaries by mobile point defects is revealed clearly from data on the strain amplitude dependence of the IF (Figure 5, Figure 6 and Figure 7). Measurements of the strain amplitude dependence of the IF are accompanied by the amplitude hysteresis, which is completely reversible in consecutive measurements down to the lowest temperatures under study. Since the work of Chambers and Smoluchowski [31] such reversible hysteresis is usually attributed to redistribution of atmospheres of mobile point defects by oscillating dislocations during measurements. When considering that the amplitude hysteresis is strongly diminished after dehydrogenation, the mobile point defects can be associated with interstitial hydrogen. The temperature range of the point-defect mobility is another point in favour of hydrogen, since other point defects (for example, vacancies or oxygen interstitials) can hardly be mobile at such low temperatures [32,33]. The data of Figure 7 allow us to discard another possible mechanism of the reversible amplitude hysteresis of the IF, namely the heating of samples by high-amplitude oscillations. In the latter case, an opposite effect of the measuring step is anticipated: the shorter measuring time for longer steps should result in less pronounced amplitude hysteresis.

The IF due to the motion of dislocations or boundaries is sensitive to concentration of point defects in the vicinity of dislocations/boundaries. The amplitude-independent IF feels the point defects situated in the core regions of dislocations/boundaries, whereas changes of point defect concentration in extended point defect atmospheres can be monitored by means of the amplitude-dependent IF. The behaviour of the amplitude-independent IF and effective Young’s modulus at the high-temperature slope of the low-temperature IF maximum on heating can be explained as a pinning stage due to an increase of the hydrogen concentration at twin boundaries (in the heat-treated states) or at grain boundaries and/or bulk dislocations (in the cold-worked state). The opposite effect (an increase of the IF and Young’s modulus defect at the same temperatures on cooling) can be regarded as a depinning stage. The dehydrogenation treatments minimize pinning, but do not extract hydrogen completely. That is why the low-temperature IF maximum (curve VA4 in Figure 3a) and amplitude hysteresis of the IF (Figure 6) still persist after vacuum anneals. The appearance of the “non-thermally activated IF maximum” in vacuum-annealed “H-free” samples [8,9,10,11,12,13] might also indicate that hydrogen is still retained in the samples.

In the heat-treated states, the more or less pronounced pinning stage is superimposed on a general trend for the IF to increase with temperature rise up to transformation temperatures, at which the IF falls due to disappearance of the martensitic phase. Since, according to the data on the amplitude dependence of the IF, the point defects (interstitial hydrogen) are mobile over the entire temperature range under study, this general trend can be associated with the decrease of hydrogen concentration in the core regions of twin boundaries caused by entropy contributions to the binding free energy of a hydrogen atom to a twin boundary [34]. According to Reference [35], in the dilute solid solutions the core concentration *C_d_ ∝ C_0_ exp* (*F_B_/kT*), where the free binding energy *F_B_ = H_B_ − TS_B_*, *C_0_* is the concentration in bulk solution, *k* the Boltzmann constant, *T* the absolute temperature, and *H_B_* and *S_B_* the binding enthalpy and binding entropy. The exponent factor includes the entropy contributions. As a result of hydrogen trapping by structural defects, the concentration of hydrogen in bulk solution *C_0_* also depends on temperature. This temperature dependence is expected only in certain temperature ranges, wherein trapping/detrapping of hydrogen by/from certain kind of traps occurs, in contrast to the entropy effect acting at all of the temperatures. Hydrogen detrapped on heating from a certain kind of traps by thermal energy tends to be redistributed between the bulk of a crystal and other kinds of traps. Thus, we can conclude that the pinning stage, observed in the IF and Young’s modulus of the heat-treated sample, is due to arriving to twin boundaries the hydrogen detrapped from other structural defects. A wide temperature range of the pinning stage points to a wide spectrum of binding energies. Bulk dislocations and/or grain boundaries are the structural defects providing wide spectra of binding energies [36]. In this connection, the accelerating rise of the IF in the cold-worked state at temperatures of the pinning stage in the heat-treated states (50–150 K, curve AR in Figure 3a) is worth noting. Furthermore, the pinning stage in the IF and Young’s modulus curves for the cold-worked state starts at temperatures (about 160 K) at which the pinning stage in the heat-treated states completes. These observations support the foregoing conclusion that bulk dislocations and/or grain boundaries are the defects responsible for the amplitude-independent IF of the cold-worked AR sample. It should also be noted that the low-temperature IF maximum is not due to a single mechanism, but it is caused by a competition of two different temperature-dependent processes. In this respect, the maximum can be regarded as a pseudo-peak. The same is true for the high-temperature IF maximum in the heat-treated sample, but its detailed analysis is beyond the scope of the present paper.

The strong dependence of the temperature position of the low-temperature IF maximum on the hydrogen content leads us to the suggestion that many of the “different” low-temperature IF peaks (Tirbonod-Koshimizu peak, P_d_, P_TWM_, P_150K_, P_200K’_, P_50K_), reported earlier for differently treated Ni-Ti-based alloys [5,6,7,8,9,10,11,12,13], may actually be the same IF maximum. The assumption of Reference [12] that P_TWM_ and P_150K_ represent the same phenomenon was the first step in this direction. In Reference [13], the P_50K_ peak, observed in the B19 martensite of Ti_50_Ni_30_Cu_20_, was regarded as a counterpart of the P_150K_ peak occurring in binary Ni-Ti alloys. Now we can add the Tirbonod-Koshimizu and P_d_ peaks to this unification. They were claimed as relaxational, but the effective modulus exhibited a pinning stage rather than relaxation (see Figure 1 in Reference [5] and Figure 10 in Reference [6]). The activation parameters in both cases were evaluated from only two experimental curves, of which only one exhibited a maximum. Nevertheless, the frequency dependence of the IF in the vicinity of the Tirbonod-Koshimizu and P_d_ peaks can hardly be ignored. It is worth to recall here that the P_150K_ peak also demonstrated frequency dependence and was initially claimed as relaxational [11], but turned out to be non-relaxation after widening the frequency range [12]. Probably, frequency dependence is inherent to the non-relaxation IF maximum under discussion as a result of the frequency dependence of pinning conditions: the higher the frequency of oscillations, the more complicated the pinning of dislocations/boundaries by arriving point defects is. This explanation is supported by the fact that the frequency dependence was registered essentially on the high-temperature slope of the IF maximum (see Figure 2 in Reference [11]).

Probably, some peaks in intentionally H-loaded samples have the same origin, for example, P_H’_ peak observed at about 40 K in Ni_47_Ti_40_Hf_10_Cu_3_ alloy doped with 1 at.% H [9,13]. Its frequency dependence, registered in a narrow frequency range, was seen as evidence of the relaxation nature, but the corresponding Young’s modulus curve revealed a pinning stage between 50 and 150 K, not a relaxation, similar to our data for the heat-treated sample.

Finally, we note that a variety of hydrogen traps can cause more than one pinning stage of dislocations/boundaries on heating. This is illustrated in Reference [12] by a two-stage decrease of the amplitude-dependent IF at T > 150 K, associated with two non-relaxation IF maxima (P_150K_ and P_200K’_). In Reference [13], these two maxima were related to “some sort of second-order phase transitions”, but there is no experimental evidence for the transitions. Two stages of pinning (150–200 and 200–300 K) are also distinguishable in our temperature IF spectra for the cold-worked AR sample.

## 5. Conclusions

(1) The martensitic transformation in the cold-worked (strain of about 40%) Ni_50.8_Ti_49.2_ alloy is only partial and very diffuse. High amplitude-independent IF in the cold-worked state is due to grain boundaries and/or bulk dislocations, rather than to twin boundaries, as is the case for heat-treated states of the alloy.
(2) Contamination of the Ni_50.8_Ti_49.2_ alloy by hydrogen strongly affects the IF and Young’s modulus of the martensitic phase.
(3) Presence of hydrogen gives rise to a non-relaxation IF maximum, whose temperature and height depend strongly on the hydrogen content.
(4) The observed non-relaxation IF maximum is formed due to a competition of two different temperature-dependent processes affecting the hydrogen concentration in the core regions of twin boundaries (after heat treatments) or grain boundaries/bulk dislocations (after cold-working).
(5) Many of the low-temperature IF peaks (Tirbonod-Koshimizu peak, P_d_, P_TWM_, P_150K_, P_200K’_, P_50K_, P_H’_), reported earlier for differently treated Ni-Ti-based alloys, have actually the same origin as the present maximum.

## Figures and Tables

**Table 1:** Hydrogen content extracted from differently treated samples.

Sample	Hydrogen Content, At. ppm		
	Extracted at 803 K	Extracted at 1073 K	Total Content
AR	11	62	73
WQ	195	102	297
VA4	9	24	33
